# BNIP3 as a new tool to promote healthy brain aging

**DOI:** 10.1111/acel.14042

**Published:** 2023-11-29

**Authors:** Kit Neikirk, Andrea G. Marshall, Monica M. Santisteban, Antentor Hinton

**Affiliations:** ^1^ Department of Molecular Physiology and Biophysics Vanderbilt University Nashville Tennessee USA; ^2^ Department of Medicine Vanderbilt University Medical Center Nashville Tennessee USA

**Keywords:** age‐related pathology, aging, BNIP3, mitochondria, neuronal

## Abstract

The article “Neuronal induction of BNIP3‐mediated mitophagy slows systemic aging in Drosophila” reveals BCL2‐interacting protein 3 as a therapeutic target to counteract brain aging and prolong overall organismal health with age. In this spotlight, we consider the roles of BNIP3, a mitochondrial outer membrane protein, in the adult nervous system, including its induction of mitophagy and prevention of dysfunctional mitochondria in the aged brain. Implications for other tissue types to reduce the burden of aging are further considered.

## GRAPHICAL ABSTRACT TEXT

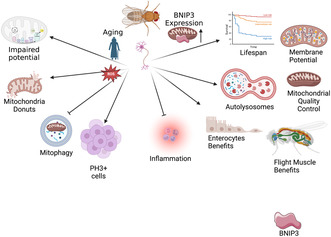

**Abbreviations**
AD: Alzheimer's Disease
BNIP3: BCL2 interacting protein 3
DOA: dominant optic atrophy
MFN2: mitofusin 2
OPA1: optic atrophy 1
PH3+: phospho‐histone H3+
ROS: reactive oxygen species

## 1 INTRODUCTION

Aging remains a global topic of interest, especially in the brain where it is linked to pathologies including Alzheimer's Disease (AD) (Grimm et al., 2016). The age‐related occurrence of AD may be exacerbated by mitochondrial dysfunction, as neurons need functional mitochondria for oxidative phosphorylation (Grimm et al., 2016). Previous studies show that mitochondria structure in the brain vary across age, with the age‐related formation of novel 3D structures including mitochondria donuts which concomitantly occur alongside cognitive decline (Hara et al., 2014). Similarly linked to age‐related mitochondrial dysfunction is the process of mitophagy, or autophagy of mitochondria, which has positive roles in removing dysfunctional mitochondria (Sun et al., 2015). The age‐related decline in mitophagy may have important implications for mitochondria structure and function, especially in age‐related pathologies like AD. Thus, an ongoing focus of the investigation has been to identify potential targets of mitophagy to slow age‐related pathology. One recent target is BCL2/adenovirus E1B 19‐kDa interacting protein 3, which is encoded by the BNIP3 gene. BNIP3 localizes at the mitochondrial outer membrane, with an N‐terminus domain that aids in binding mitochondria to autophagosomes through ATG8/LC3 regions (Schmid et al., 2022). As a proapoptotic protein, past research has demonstrated that BNIP3 can induce autophagy, and mitophagy (Gustafsson, 2011), yet the potential of BNIP3 as a therapeutic to slow aging had remained poorly elucidated until now.

**FIGURE 1:**
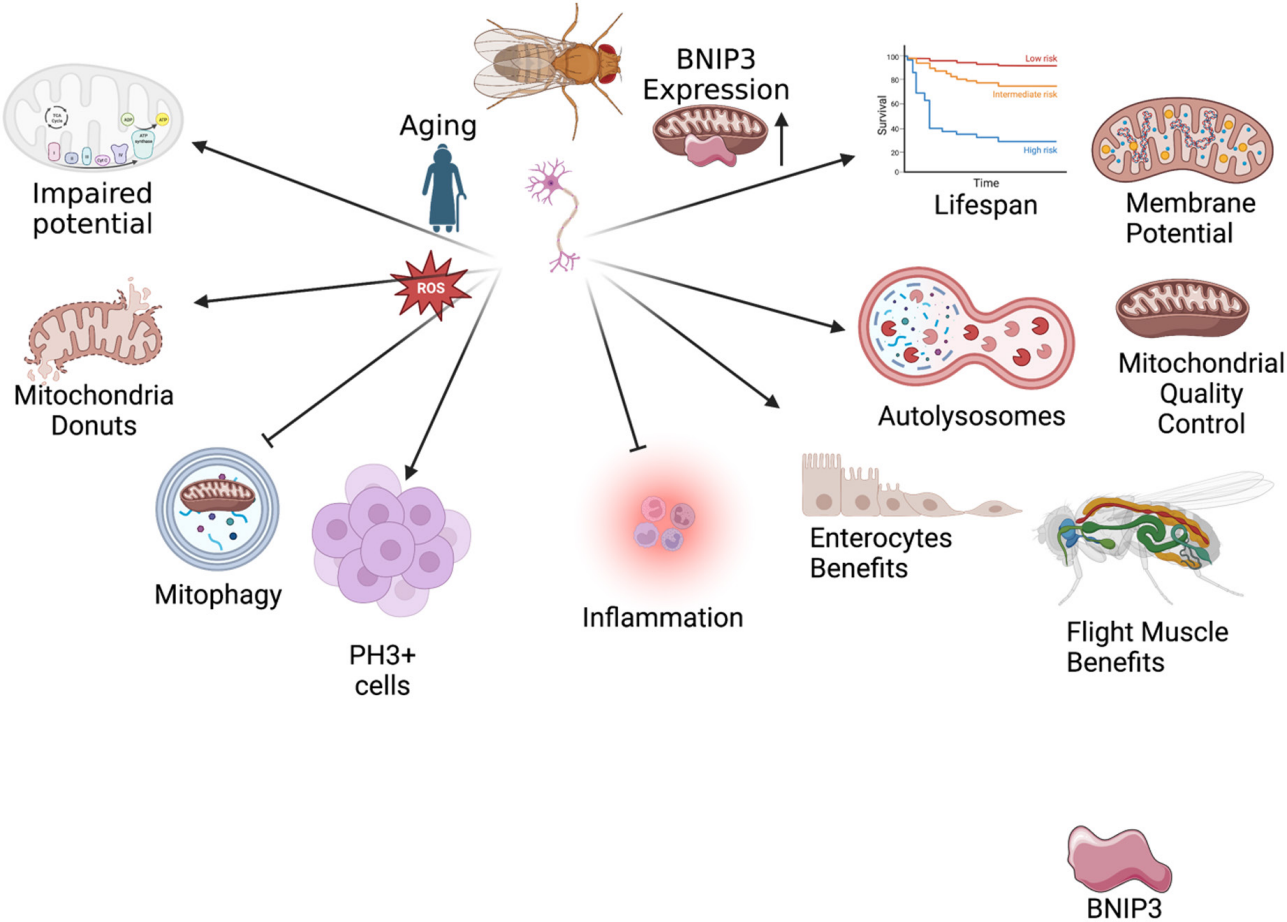
Showing pathways across aging and altered pathways with the addition of BNIP3.

Schmid and colleagues find that decreased mitochondrial autophagy results in the accumulation of dysfunctional mitochondria across aging in Drosophila brain tissue. The authors show that activation of BNIP3 can reverse this by increasing mitophagy with a concomitant uptick in the clearance of mitochondria, resulting in improved homeostasis in other regions, and organismal lifespan (Schmid et al., 2022). To come to these conclusions, Schmid et al., employed a variety of techniques. While they did not consider mitochondrial 3D ultrastructural changes across aging, they noted an accumulation of mitochondrial content concomitant with neuronal loss, as determined through immunostaining and mtDNA levels. Through neuronal‐specific BNIP3 induction, mitochondrial content decreased, and homeostasis was restored due to mitophagy, as confirmed through an increased quantity of autolysosomes through GFP‐mCherry staining for ATG8a. Importantly, *Drosophila* lifespan was extended, potentially by improving health span markers such as physical activity in an autophagy‐dependent manner. The authors also show that these pro‐lifespan effects are conferred to flight muscle and enterocytes are dependent on neuronal‐autophagy across aging (Figure 1).

To date, the role of BNIP3 in the central nervous system has mostly been investigated in the context of stroke and malignant glioblastoma multiforme. BNIP3 expression in the brain is induced by hypoxia, such as in the core of glioblastomas, but also during cerebral ischemia (Schmidt‐Kastner et al., 2004; Zhang et al., 2007). BNIP3 actually promotes neuronal cell survival, thus it is not surprising that its expression is neuroprotective in stroke (Shi et al., 2014) but detrimental in glioblastoma (Burton et al., 2013). Interestingly, BNIP3 may play different roles in glia versus neuronal cells. BNIP3 expression has been suggested to protect against neuroinflammation (Iyalomhe et al., 2017), potentially by promoting microglia homeostasis (Lei et al., 2018) and preventing NLRP3 inflammasome activation in microglia (Gong et al., 2020). On the other hand, BNIP3 is detrimental in oligodendrocyte precursor cells, since it increases their vulnerability to subsequent ischemic events, and white matter damage (Guan et al., 2022). In astrocytes, BNIP3 prevents cell death (Burton et al., 2006), and was recently identified to be downregulated in human iPSC‐derived astrocytes from patients with frontotemporal dementia type 3 (Chandrasekaran et al., 2021), indicating an additional therapeutic possibility for BNIP3. The contribution of BNIP3 to cognitive decline has not yet been uncovered, thus it would be interesting to explore its role in cognitive function during healthy and abnormal brain aging.

Past results have demonstrated that skeletal muscle BNIP3 increases with aging and interestingly was associated with healthy muscle aging, as loss of BNIP3 resulted in inflammation in a mitochondrial‐dependent manner (Irazoki et al., 2022). However, Schmid et al., advanced this understanding to show that activation of neuronal BNIP3 can begin to reverse age‐related changes. BNIP3 was also noted to restore age‐related decline of membrane potential, as observed through Tetramethylrhodamine, ethyl ester (TMRE), including in other tissue types such as skeletal muscle (Schmid et al., 2022). Notably, long‐lived animals including naked mole rats and bats can potentially extend lifespan through mechanisms that protect membrane depolarization (Vyssokikh et al., 2020), highlighting the consequence of membrane potential across aging. Interestingly, past studies in mice show that loss of BNIP3 results in larger mass mitochondria in the liver, yet these mitochondria had abnormal structure and exhibited loss of membrane potential (Glick et al., 2012). Loss of membrane potential can cause factors including increased reactive oxygen species (ROS), inflammation, and mitochondrial swelling to impair mitochondrial transport (Kokkinopoulos et al., 2013; Safiulina et al., 2006). BNIP3‐dependent reversal of mitochondrial dysfunction suggests that BNIP3 may also play roles in remodeling mitochondria and maintaining healthy populations in mechanisms beyond only mitophagy, which may explain how BNIP3 has beneficial roles in other organ systems.

BNIP3 was also shown to reduce phospho‐histone H3+ (PH3+) in midguts by Schmid et al. Generally, the role of PH3+ cells is unclear in aging; while PH3+ cells are important for cell proliferation in aging models, hyperproliferation occurs concomitantly with rising PH3+ levels, which can be stopped by reducing PH3+ levels, thus extending life span (Biteau et al., 2010). Notably, while the research remains ongoing, this protective effect of BNIP3 appears to be independent of mitochondria and may arise from BNIP3's apoptotic effects. BNIP3 may also function in PINK1‐dependent manners to promote mitophagy (Schmid et al., 2022). Together, these offer avenues that need to be explored further in how BNIP3 functions.

One important avenue of future research is understanding regulators of BNIP3 levels across aging, beyond exclusively *Drosophila* models. Notably, ROS levels increase can cause a concomitant rise in BNIP3 expression (Sun et al., 2022). Indeed, resistance to BNIP3‐mediated apoptosis is regulated by mitochondrial antioxidant manganese superoxide dismutase levels (Lee et al., 2015), suggesting that the ability of different cell types to respond to BNIP3 levels is partially dictated by ROS that are generated across aging. Thus, logically, some tissue types including skeletal muscle show an age‐related increase in BNIP3 levels (Huang et al., 2020), yet in other tissue types, such as cochlea, there is an age‐related loss (Oh et al., 2020). The causal relationship between these factors must further be explored, as it is unclear if the uptick in BNIP3 acts as a protective mechanism against increased ROS and inflammation, or if an age‐related generation of ROS results in BNIP3 levels being correlated with age (Irazoki et al., 2022).

Interestingly, past studies have implicated BNIP3 in the progression of Dominant optic atrophy (DOA), a disease that arises due to mutations in OPA1, an important fusion protein in the mitochondrial dynamics (Moulis et al., 2017). Notably, impairments in OPA1 levels occur concomitantly with reduced BNIP3 levels, with the latter being restored in an ROS‐dependent manner (Moulis et al., 2017). This suggests that impaired mitophagy may occur alongside mitochondrial structure abnormality, yet it remains unclear if BNIP3‐levels modulate those of OPA1. While the relationship between aging and OPA1 requires further elucidation, current studies suggest that across aging, fusion proteins including OPA1 are increased (Son et al., 2017). However, BNIP3 clearly has differential effects in tissue, as does OPA1, making it difficult to elucidate if simply increasing the expression of both proteins can undo the age‐related decline of mitochondria, even in neuron‐specific models. Similarly, in the context of MFN2, another mitochondrial protein which may have altered expression across aging, deficiency of MFN2 can cause ROS to enact BNIP3 pathways to avoid mitochondrial dysfunction (Sebastián et al., 2012). This suggests that BNIP3 can serve as an alternative mechanism to maintain mitochondrial structure in the dysregulation of dynamic proteins, but it remains unclear if this mechanism also is activated in response to other mitochondrial or cristae proteins.

Notably the human brain, similar to skeletal muscle, shrinks during aging (Schmid et al., 2022); therefore, BNIP3 could be a target in sarcopenia if it reduces a loss of mass due to the maintenance of mitochondrial populations. Past literature shows that in skeletal muscle BNIP3 levels actually increased in aging, so it remains unclear if this increase may serve as a protective mechanism against sarcopenia (Huang et al., 2020). The authors show flight muscle mitochondrial dynamics are also restored through neuronal‐specific induction, so it is unclear if this same mechanism may alter mass and strength in sarcopenia.

Several future avenues clearly need to be explored following this study. There is a clear need to study if autophagy machinery and their ultrastructure, in addition to that of mitochondria, are altered through modulation of BNIP3 activity (Neikirk et al., 2023). Equally, important to understand 3D structure of mitochondrial phenotypes and their associated frequency which may offer clues as to how mitochondria are modulated (Glancy et al., 2020). Beyond this, while Schmid and colleagues evaluate the effects of one‐week or two‐week periods which showed no differences, an adjusted BNIP3 induction period must be considered (Schmid et al., 2022). Finally, the context of potential co‐factors of BNIP3 and pathways it acts in remain poorly elucidated in this study, as past studies show that some mechanism of action of BNIP3 may be reliant on synergistic effects of Nix, at least in cardio‐myocytes (Dorn, 2010).

Beyond these, factors while the authors show BNIP3 increases lifespan independent of changes in diet (Schmid et al., 2022), it remains unclear if BNIP3 can also increase longevity in disease states or with alterations in diet and lifestyle. Given that autophagy is linked to age‐onset neurodegeneration, it is logical that BNIP3 may also reduce the incidence of age‐related cognitive decline. Beyond this, fasting causes an increase in BNIP3 in the liver (Glick et al., 2012), but it is unclear if these also have effects on neuronal expression. Contrastingly treadmill exercise can reduce BNIP3 levels in cardiac myocytes which may be beneficial in the context of cardiac apoptosis (Arabzadeh et al., 2022).

In conclusion, this study represents an important broadening of understanding regarding the functions of BNIP3. Still, it remains unclear if BNIP3 expression can also offer antiaging effects in other tissue types, as the research by Schmid et al., suggests that only BNIP3 induction specific to neurons has antiaging effects to promote skeletal muscle and intestinal health. Indeed, it is not yet clear why ubiquitous induction may not have these same effects and suggest that BNIP3 in certain systems can have contrastingly negative effects in apoptotic pathways. Notably, Schmid et al., show that pro‐lifespan effects of BNIP3 do not occur with nonspecific induction to neurons, indicating that apoptotic pathways potentially play tissue‐dependent roles in neurons (Schmid et al., 2022). A striking difference between BNIP3 expression in the brain versus skeletal muscle is its cellular localization. In the brain, BNIP3 has relatively low expression and is primarily localized to the nucleus, whereas in skeletal muscle, it is robustly expressed and localized throughout the cell (Burton et al., 2006). Past research on BNIP3 in the context of cancers suggest that the different cellular localization may relate to its signaling pathways (Shaida et al., 2008). Thus, it remains to be investigated whether these differences in cellular localization affect the balance between pro‐ and antiapoptotic pathways, and future research is needed to uncover how inter‐organ signaling may improve life‐span.

## AUTHOR CONTRIBUTIONS

Conceptualizing: Antentor O. Hinton, Jr., Writing; Research; Drafting; Editing: Kit Neikirk, Andrea G. Marshall, Monica M. Santisteban, Antentor O. Hinton, Jr., Final Approval: Antentor O. Hinton, Jr.

## FUNDING INFORMATION

The UNCF/Bristol‐Myers Squibb E.E. Just Faculty Fund, Career Award at the Scientific Interface (CASI Award) from Burroughs Welcome Fund (BWF) ID # 1021868.01, BWF Ad‐hoc Award, NIH Small Research Pilot Subaward to 5R25HL106365‐12 from the National Institutes of Health PRIDE Program, DK020593, Vanderbilt Diabetes and Research Training Center for DRTC Alzheimer's Disease Pilot & Feasibility Program. CZI Science Diversity Leadership grant number 2022‐ 253529 from the Chan Zuckerberg Initiative DAF, an advised fund of Silicon Valley Community Foundation (AHJ). The funders had no role in the study design, data collection and analysis, decision to publish, or preparation of the manuscript.

## CONFLICT OF INTEREST STATEMENT

The authors have no Conflicts of Interest to declare.

## Data Availability

Data sharing is not applicable to this article as no new data were created or analyzed in this study.
